# Evaluating the Diagnostic Accuracy of Impression Cytology for Conjunctival Lesions: A Comparative Study with Histopathology 

**DOI:** 10.30699/ijp.2024.2025786.3284

**Published:** 2025-01-10

**Authors:** Fatemeh Eslami, Hamidreza Ghasemibasir, Sara Alipour, Ramin Mansouri

**Affiliations:** 1 *Department of Ophthalmology, School of Medicine, Hamadan University of Medical Sciences, Hamadan, Iran*; 2 *Department of Pathology, School of Medicine, Hamadan University of Medical Sciences, Hamadan, Iran*; 3 *Student Research Committee, Hamadan University of Medical Sciences, Hamadan, Iran*

**Keywords:** Conjunctival lesions, Impression cytology, Pathology

## Abstract

**Background & Objective:**

Conjunctival lesions have a wide range of histological manifestations that are difficult to distinguish clinically. The gold standard for diagnosis of these lesions is the pathological examination, a costly and invasive procedure that may also adversely affect patients. Therefore, clinical researchers seek less invasive, inexpensive, and easier methods to detect conjunctival lesions. This study aims to compare the accuracy of impression cytology with pathology in patients referred to Farshchian Hospital in Iran.

**Methods:**

In this descriptive/cross-sectional study, 64 patients with conjunctival lesions were selected from patients referred to Farshchian Hospital in Hamedan in 2021. A cytology specimen was obtained from the patients and sent to the laboratory. The diagnostic accuracy of this method was compared with pathological results (gold standard). Data were analyzed by SPSS 16 software.

**Results:**

The mean age of patients was 54.47 ±16.94 years; 70.3% were male, and 29.7% were female. In the cytologic and pathologic examination, 28.1% of the specimens showed dysplasia, and 71.9% were non-dysplastic. The sensitivity, specificity, positive predictive value, negative predictive value, and accuracy of the cytologic impression compared to the pathologic methods were 91.30%, 77.78%, 91.30%, 77.78%, and 87.5%, respectively. A positive and significant correlation was observed between pathological and cytological diagnosis scores (r=0.825, P-value<0.001).

**Conclusion:**

In conjunctival lesions, impression cytology may be a relatively accurate and sensitive procedure that can distinguish dysplastic from non-dysplastic conjunctival lesions.

## Introduction

The conjunctiva is a visible tissue exposed to sunlight, making it easy to diagnose tumors and related lesions in their early stages (1). Conjunctival tumors encompass a wide range of neoplasms originating from different sources and exhibiting varying degrees of malignancy, ranging from benign to more aggressive neoplasms. (2). Exposure to sunlight, human papillomavirus (HPV) subgroups 6, 11, and 16, and HIV infection are the primary factors linked to conjunctival epithelial lesions. Also, Men are more likely to develop squamous cell dysplasia/carcinoma due to their increased exposure to sunlight while working outside the home (3, 4). Factors that can contribute to conjunctival surface dysplasia include exposure to petroleum products, cigarette smoking, and certain chemicals like trifluoridine, arsenic, and beryllium (5). Vitamin A deficiency, light pigmentation of scalp and eyebrow hair, family origin from Australia, New Zealand, or the United Kingdom, and immunosuppressive diseases may also play a role. Dysplasia/squamous cell carcinoma typically presents in individuals who are in their 60s and 70s (6). 

Ophthalmologists and ophthalmic pathologists frequently encounter ocular surface tumors with diverse clinical manifestations (7). Clinically, these lesions typically appear as reddish-gray or gelatinous masses with prominent vascular tufts. They are mostly found in the interpalpebral area near the nasal or temporal limbus (8). Various types of ocular surface neoplasms are categorized based on where they originate (9). Nonmelanocytic cancers, such as squamous papilloma, conjunctival-corneal intraepithelial neoplasia, and squamous cell carcinomas (SCCs), start to grow in the squamous epithelium. Meanwhile, melanocytic lesions include nevi, primary acquired melanomas, and malignant melanomas. It's important to rule out malignancy in cases involving rapid growth, color changes, and chronic inflammation (10). The mutation of the p53 gene is a frequent occurrence in the development of neoplasia (6). Ocular surface squamous neoplasia (OSSN) is a common premalignant lesion on the eye's surface. It can range from mild to severe dysplasia and, in some cases, may develop into invasive squamous cell carcinoma (SCC). Although the appearance of a lesion may indicate OSSN, a tissue biopsy is needed to confirm the diagnosis. This is because distinguishing between the different stages of OSSN is challenging, even for experienced clinicians using slit-lamp biomicroscopy, with an accuracy rate of 40%(11). A major concern with tissue biopsy is the risk of unnecessary surgical procedures for patients. To mitigate this, it is recommended that appropriate diagnostic tests be conducted beforehand (12).

To further analyze and diagnose various ocular surface disorders, samples are taken from the eye's surface and processed using appropriate methods, performed in exfoliative cytology and impression cytology. Impression cytology (IC) was first developed for diagnosing dry-eye status but is now used to diagnose neoplasia and other disorders (13). IC is especially helpful in evaluating suspicious ocular surface lesions, as repeated surgical biopsies may cause complications such as scarring, lid deformity, limbal stem cell deficiency (LSCD), and discomfort for the patient (11, 14, 15). Cytological evaluation can differentiate between inflammatory and neoplastic lesions, guiding further diagnostic tests and treatment before invasive techniques are utilized (16). Recently, IC has been used to investigate gene and protein expression in conjunctival cells. This has led to identifying novel diagnostic biomarkers and a better understanding the mechanisms underlying ocular surface disease (17). Performing this procedure has several advantages. Firstly, it only requires the use of local anesthesia. Additionally, it maintains cell-to-cell contact and does not alter cell morphology. Furthermore, any microscopy, PCR, or immunoblotting can be used to analyze cytology impression samples (18-20). A small amount of topical anesthesia is applied to prepare for the procedure. Then, forceps are applied to apply a membrane with submicroscopic pores, like MF-Millipore, onto the affected eye's surface. The membrane is often pre-cut in various shapes and sizes to aid in the processing and orientation of the lesion. Then, to collect a sample, press a membrane firmly against the area using a swab or solid rod for a few seconds. Then, use forceps to peel off the membrane. A more severe stage is considered if various types of atypical cells are present in the same specimen (20). Ocular cytology specimens are small, and obtaining a repeat biopsy can be difficult. Appropriate handling and triaging for ancillary testing is important (21).

Even with this, impression cytology also has limitations, such as the method only allowing for the collection of samples from the surface of cells and the necessity for an experienced cytologist (22). This non- or minimally invasive biopsy technique can be applied to the conjunctiva, cornea, and limbal area for both diagnosis and follow-up after treatment of tumors.

This study aims to compare the accuracy of impression cytology with pathology as the gold standard for diagnosis in patients referred to Farshchian Hospital in Iran. We will evaluate the sensitivity and specificity of impression cytology, particularly in detecting dysplastic and neoplastic lesions.

## Material and Methods

This cross-sectional study was conducted in 2021on all 64 samples suspicious for neoplastic or dysplastic changes in the ocular surface during clinical examination by an expert ophthalmologist at the eye clinic of Farshchian Hospital in Hamedan, which were referred to the Pathology Department of Farshchian Hospital in Hamedan.

This study (IR.UMSHA.REC.1397.186) received approval from the Medical Center Institutional Review Board (IRB), and all procedures were carried out according to their guidelines. This study was conducted strictly according to the Clinical Practice Guidelines and ethical guidelines outlined in the Declaration of Helsinki and received approval from the IRB. All participants provided informed consent before their inclusion in the study.

Sample size was estimated to be 60 people based on 95% sensitivity and using the results of Tole* et al.*'s study (14) and according to the sample size calculation formula to determine the sensitivity and specificity of diagnostic methods and compare the results of impression cytology and pathology tests. In this study 64 people were investigated. sample size nbased on sensitivity=Z1-α/22×SN×1-SNL2×Prevalence

In this formula, (Z_1-alpha/2_) represents the critical value from the standard normal distribution corresponding to the significance level, (S_N_) refers to the sample proportion, and (L) represents the margin of error.

The criteria for entering the study were having neoplastic or dysplastic lesions of the eye surface and giving contest from the patients before preparing a cytologic sample from the eye conjunctiva, using the impression method, after providing the patients with clear information.

The exclusion criteria were set to ensure the fairness and integrity of the study. Patients with corneal ulcers were excluded, as well as those who declined to participate in the study. It's important to note that there was no charge for the preparation of the sample, ensuring that financial constraints did not influence participation.

After obtaining written consent from the patients, using sterile cellulose acetate filter paper of Sartorius company with pore size 0.22 μm (micrometer), a cytology sample was prepared from the eye conjunctiva by impression method and sent to the pathology laboratory after placing it in a special preservative solution. At the same time, a biopsy sample was routinely prepared from the eye surface lesion and sent for pathology. All cytology samples were sent to a laboratory and examined by an experienced pathologist to avoid bias. The pathology assistant also reviewed the pathology test of the tissue samples without knowing the cytology results. The slide was labeled and numbered, and then it was stained with periodic acid-Schiff and counterstained with hematoxylin and eosin. The mounted slide was first examined under the microscope with × ten 10-high power field (HPF). After localization, cells were then analyzed with × 40 HPF magnification.

It is necessary to explain that in the present study, the scoring method is based on nuclear changes (score 0-2), nucleus size (score 0-1), hyperchromasia (score 0-1), and nuclear polymorphism (score 0-1). Nucleus-to-cytoplasm ratio (score 0-1), nuclear membrane (score 0-1), rough chromatin (score 0-1), nucleus (score 0-1), mitosis (score 0-1), binucleate or multinucleate cell (score 0-1), syncytial pattern (score 1-0), overlapping and crowding (score 0-1) and inflammatory infiltration (score 0-1) were performed.

We have categorized squamous cell abnormalities into four groups using a modified version of the Bethesda system in gynecology. These groups are: 

1) Atypical squamous cells (ASCs) 

2) Low-grade squamous intraepithelial lesions (LSILs), which include squamous papilloma and mild dysplasia 

3) High-grade squamous intraepithelial lesions (HSILs), which include moderate to severe dysplasia and carcinoma in situ (CIS) 

4) Squamous cell carcinoma (SCC)(23).

The results of cytology and pathology were analyzed after registering in the checklist designed by a statistical consultant with SPSS version 16 software (SPSS Inc., Chicago, Ill., USA). In the data analysis, the qualitative data's descriptive information was expressed in tables, graphs, ratios, and percentages. The Kolmogorov-Smirnov test evaluated the normality of the data. Fisher's exact test and Chi-square were used to compare nominal and rank qualitative variables. Student's t-test and one-way analysis of variance were used to compare quantitative variables between two groups and more than two groups. The sensitivity, specificity, and positive and negative predictive value of cytology diagnosis compared to pathology (standard) were calculated manually using standard formulas. All analyses were performed at a 95% confidence level, and a P-value less than 0.05 was considered significant.

## Results

This study, included 64 samples of eye conjunctival lesions. Regarding gender, 45 patients (70.3%) were male, and 19 (29.7%) were female. The average age of patients was 54.47±16.94 years, ranged from 20 to 95 years. The age group of 35 to 40 years showed highest frequency.

In patients suspicious for conjunctival dysplastic lesions, 28.1% were non-dysplastic, and 71.9% were dysplastic, confirmed cytologically and histopathol-ogically. In the comparison of cytologic and pathologic results for conjunctival lesions based on age and gender, no statistically significant differences were found, except for pathology results, which showed more severe dysplasia and SCC in men than in women (P < 0.05). 

Out of the 46 patients who were diagnosed with dysplastic lesions of the eye conjunctiva by pathology, 42 (91.3%) were diagnosed with dysplasia (true positive), and 4 (8.7%) were diagnosed as non-dysplastic (false negative) by cytology. 

Out of 18 patients who were diagnosed with non-dysplastic eye conjunctival lesions by pathology method, 14 (77.8%) of them were not dysplastic (true negative), and 4 (22.2%) were dysplastic (false positive) by cytology were diagnosed.

Impression cytology's sensitivity, specificity, positive predictive value, and negative predictive value in diagnosing dysplastic and non-dysplastic lesions were 91.30%, 77.8%, 91.30%, and 77.8%, respectively. The detection accuracy was also 87.5%.

The summation of 13 parameters used in diagnosing dysplasia in cytology slide examinations yields a total cytology score, ranging from 0 to 14. As this score increases, the degree of dysplasia also increases. According to the post hoc Tukey test results, the mean cytology score was significantly lower in patients without dysplasia compared to those with atypical aquamous aells (ASC). Similarly, the ASC score was lower than that of low-grade squamous intraepithelial lesion (LSIL), and LSIL had a lower score than high-grade squamous intraepithelial lesion (HSIL) and squamous cell carcinoma (SCC) (P = 0.001) (Table 1). 

A statistically significant difference was observed between the frequency of cytologic findings in each of the four cytolog groups (*P*<0.05) (Table 2).

**Fig. 1:**
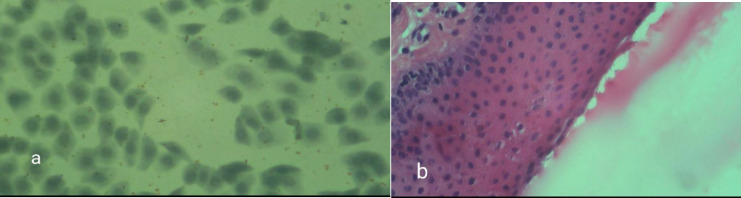
a) cytology sample showing no dysplasia, b) Pathology sample showing no dysplasia

Of 18 cases that did not show dysplasia by pathology, 14 were correctly diagnosed without dysplasia by cytology (77.7% agreement of results) (Figure 1).

**Fig. 2:**
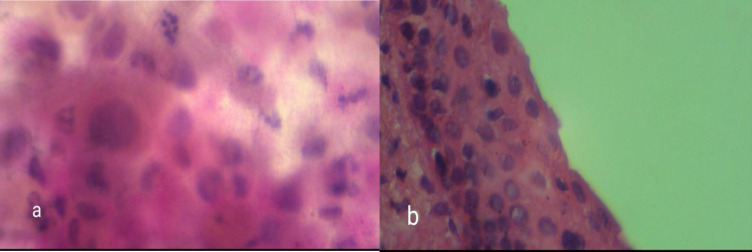
a) cytology sample showing mild dysplasia, b) Pathology sample showing ASC

Four of six cases with mild dysplasia diagnosed by pathology were correctly diagnosed as ASC dysplasia by cytology (66.6% matching results) (Figure 2).

**Fig. 3:**
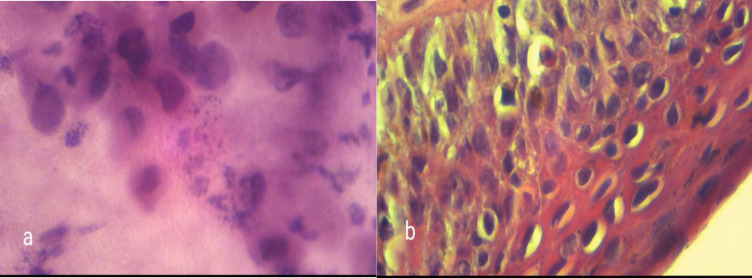
a) cytology sample showing moderate dysplasia, b) Pathology sample showing LSIL

Four of nine cases with moderate dysplasia diagnosed by pathology were correctly diagnosed as LSIL dysplasia by cytology (44.4% matching results) (Figure 3).

**Fig. 4:**
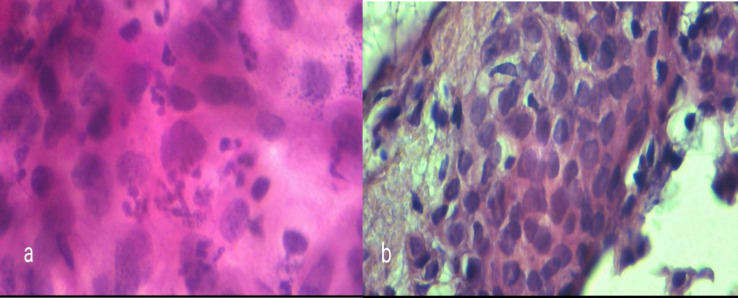
a) cytology sample showing severe dysplasia, b) Pathology sample showing SCC

Of the 31 cases with severe dysplasia or SCC by pathology method, 19 were correctly diagnosed as SCC & HSIL dysplasia by cytology (61.3% matching results) (Table 3) (Figure 4). The Kappa agreement coefficient between the results of cytology and pathology was 51% (*P*<0.001), And according to the two-mode rating method, dysplastic and non-dysplastic was 69% (*P*<0.001).

**Fig. 5:**
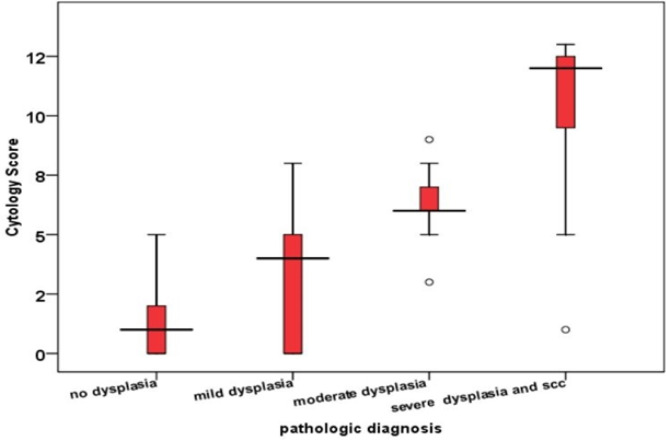
Comparison of the distribution of frequency of the patients' cytology score-based pathology results

As can be seen in the box plot diagram, the median cytology score is lower in samples without dysplasia and higher in severe dysplasia compared to the pathology result. Based on the Kruskal-Wallis non-parametric statistical test results and Tukey's post hoc test, no statistically significant difference was observed between the mean cytology score in patients diagnosed with pathology without dysplasia and mild dysplasia. However, the average cytology score in patients diagnosed with moderate and severe dysplasia pathology was significantly higher than mild dysplasia and no dysplasia (*P*<0.05) (Figure 5)

According to Spearman’s coefficient test result, a positive and significant correlation was observed between pathological and cytological diagnosis scores (r=0.825, *P*<0.001).

**Table 1:** Frequency and mean score of the cytology according to the final result of cytology

P-value	Standard deviation	Mean of cytology score	Number	Cytology
>0.001	0.89	0.73	18	No dysplasia
	1.46	5.14	15	ASC
	1.80	9.17	12	LSIL
	1.27	12.05	19	SCC & HSIL
	4.42	6.70	64	Total

**Table 2:** Frequency of the cytological findings in each of the four cytology groups

P-value	Total (%)	SCC& HSIL (%)	LSIL (%)	ASC (%)	No dysplasia (%)	Cytology
Nuclear changes						
0.001>	17 (100) 17 (100) 30 (100)	0 (0) 1 (5.9) 18 (60)	0 (0) 1 (5.9) 11 (36.7)	0 (0) 14 (82.3) 1 (3.3)	17 (0) 1 (5.9) 0 (0)	Unchanged Limited changes Released changes
Nucleus size						
0.001>	23 (100) 41 (100)	1 (4.3) 18 (43.9)	1 (4.3) 11 (26.8)	3 (13) 12 (29.3)	18 (78.4) 0 (0)	Less than 2 times More than 2 times
Hyperchromasia						
0.001>	18 (100) 46 (100)	0 (0) 19 (41.3)	0 (0) 12 (26.1)	1 (5.6) 14 (30.4)	17 (94.4) 1 (2.2)	Negative Positive
Nucleus polymorphism						
0.001>	23 (100) 41 (100)	0 (0) 19 (44.2)	2 (9.5) 10 (23.3)	2 (9.5) 13 (30.2)	17 (81) 1 (2.3)	Negative Positive
Nucleus to cytoplasm ratio						
0.001>	38 (100) 26 (100)	0 (0) 19 (73.1)	6 (15.8) 6 (23.1)	14 (36.8) 1 (3.8)	18 (47.4) 0 (0)	Less than 1.1 More than 1.1
Nuclear membrane						
<0.001	35 (100) 29 (100)	1 (2.9) 18 (62.1)	6 (17.1) 6 (20.7)	10 (28.6) 5 (17.2)	18 (51.4) 0 (0)	Regular Irregular
Coarse chromatin						
<0.001	28 (100) 36 (100)	1 (3.6) 18 (50)	1 (3.6) 11 (30.6)	8 (28.6) 7 (19.4)	23 (64.18) 0 (0)	Negative Positive
Mitosis						
<0.052	55 (100) 9 (100)	10 (18.2) 9 (100)	12 (21.8) 0 (0)	15 (27.3) 0 (0)	18 (32.7) 0 (0)	Negative Positive
Nucleolus						
0.57	60 (100) 4 (100)	15 (25) 4 (100)	12 (20) 0 (0)	15 (25) 0 (0)	18 (30) 0 (0)	Negative Positive
Bi- or multinucleated cell						
0.013	34 (100) 30 (100)	6 (17.6) 13 (43.3)	6 (17.6) 6 (20)	8 (23.5) 7 (23.3)	14 (41.3) 4 (13.3)	Negative Positive
Syncytial pattern						
<0.001	34 (100) 30 (100)	0 (0) 19 (63.3)	3 (8.8) 9 (30)	15 (44.1) 0 (0)	16 (47.1) 2 (6.7)	Negative Positive
Overlap and crowding						
0.001	32 (100) 31 (100)	0 () 19 (61.3)	4 (21.1) 8 (25.8)	14 (42.4) 1 (3.2)	15 (45.5) 3 (9.7)	Negative Positive
Infiltration						
0.006	40 (100) 24 (100)	4 (10) 15 (62.6)	7 (17.5) 5 (20.8)	13 (32.5) 2 (8.3)	16 (40) 2 (8.3)	Negative Positive

**Table 3:** Frequency of cytological and pathological results of conjunctival lesions of the eye in subgroups

Total	Severe dysplasia and SCC	Moderate dysplasia	Mild dysplasia	No dysplasia	Cytology
18	1	1	2	14 (77.7%)	No dysplasia
15	3	4	4 (66.6%)	4	ASC
12	8	4 (44.4%)	0	0	LSIL
19	19 (61.3%)	0	0	0	HSIL &SCC
64	**31**	**9**	**6**	**18**	**Total**

## Discussion

Our present study examines the diagnostic accuracy of impression cytology in eye conjunctival lesions compared to pathology, and There was a positive and significant correlation between pathological and cytological diagnosis scores, so IC can help in the early diagnosis of lesions and the detection of premalignant stages of SCC, which is crucial for successful treatment. Also, it can be valuable in clinical decision-making, OSSN management, and monitoring treated cases (24). 

OSSN is a condition that results from the interaction of several risk factors (25). In a study conducted by Roland Hӧllhumer* et al.* to investigate the demographics, clinical manifestations, and risk factors of ocular surface squamous neoplasia in a tertiary hospital in South Africa, the mean age was 44 years with equal gender distribution in the patients (26). Contrary to our study's results, no significant difference was observed in the cytology and pathology of eye conjunctival lesions based on age and gender, except for the pathology result where men had more severe dysplastic and SCC than women. Additional risk factors were not investigated and should be evaluated.

A study reported finding a similar cytomorphological spectrum of OSSN in elderly patients, with a higher incidence of high-grade dysplasia and invasive SCC compared to younger patients (27).

In the present study, 28.1% of the patients with dysplastic lesions and 71.9% were non-dysplastic in patients suspected of conjunctival dysplasia lesions by cytology and pathology methods. The sensitivity, specificity, positive predictive value, and negative predictive value of impression cytology in diagnosing dysplastic and non-dysplastic lesions were 91.30%, 77.78%, 91.30%, and 77.78%, respectively, and its accuracy was 87.5% and r=0.825. In a study conducted by Fatima* et al.* from India in 2019 regarding the correlation between Impression Cytology and histopathology of benign and malignant tumors, on 100 surgical samples, 78% of the samples were benign, and 22% were malignant. About 85% of benign and 73% of malignant samples were correctly diagnosed by cytology. A significant correlation between cytology and pathology was observed in benign and malignant lesions (*P*<0.05). The Pearson correlation coefficient between Impression Cytology and pathology in benign cases had r=0.872, the accuracy of diagnosis was 87.2%, and in malignant cases, it had r=0.746, and the accuracy of diagnosis was 74.6% (28). The two studies show consistent results regarding diagnostic accuracy and the correlation coefficient between Impression Cytology and pathology. Another study conducted by Vinod* et al.* compared the diagnostic method of impression cytology to pathology (golden standard); its sensitivity ranges between 84% and 92.1%, specificity between 20% and 94%, and positive predictive value between 89.7%. Up to 92%, the negative predictive value was between 25% and 94%, and its accuracy ranged between 81.25 and 83.72%. The present study's findings in the sensitivity, specificity, positive predictive value, and negative predictive value of impression cytology in diagnosing dysplastic and non-dysplastic lesions are consistent with our study (29).

The average cytology score in four groups without dysplasia, ASC, LSIL, HSIL & SCC, was 0/73, 5.14, 9.17, and 12.05, respectively, significantly higher in patients with severe dysplasia and SCC. It was more than LSIL dysplasia, and LSIL dysplasia was more than ASC dysplasia; in our study, the agreement between cytology and pathology results were: severe dysplasia (61.3%), moderate dysplasia (44.4%), mild dysplasia (66.6%) and cases without dysplasia (77.7%). Also, in a study which is conducted in 2022, When the cytology results were compared to the histologic outcomes for patients diagnosed with squamous neoplasia, SCC from cytology had the highest rate of association with histology (91.67%), followed by HSILs (45.5%), ASCs (42.9%), normal (33.3%), and LSILs (21.4%). This series showed that SCC is the biggest category and has the highest correlation rate with histology (30). In 2008, Tananuvat* et al.* conducted a study on the role of impression cytology in diagnosing eye conjunctival neoplasia. The study found that impression cytology had a high correlation with histological findings in diagnosing SCC (91.7%), moderate correlation with HSILs (45.5%), low correlation with LSLs (4.4%), ASC (42.9%), and normal (33.3%) cases (31). All of the studies are different from our conclusion.

In comparing the cytological diagnosis ranking (no dysplasia, dysplasia, ASC, LSIL, and HSIL) with the pathological grading of conjunctival lesions (no dysplasia, mild, moderate, and severe dysplasia), the Kappa agreement coefficient between the two methods was approximately 51%. When using the two-category rating of dysplastic vs. non-dysplastic, the coefficient was 69%.

A 2017 study by Vinod* et al.* in India investigated the correlation between impression cytology and histopathology for diagnosing ocular surface squamous neoplasia (OSSN). The study included 42 patients (43 eyes), and the results showed a Kappa agreement coefficient of 83.72%, which was higher than ours (32).

In another study from 2020 in Iran, titled “Impression cytology for detection of clinically suspected ocular surface disorders over 14 years in a referral center in Iran,” histopathologic results were available for 22 eyes and were well-correlated with the corresponding IC results (Cohen’s Kappa coefficient = 0.86) (18). Impression cytology is a straightforward, non-invasive method that can aid in the diagnosis and monitoring of ocular surface disorders. It is useful for identifying individuals who are likely to develop symptoms and for evaluating the severity of cytological changes in symptomatic patients (19, 33, 34).

Impression cytology can only assess superficial cells and cannot sample deep lesions or invasive diseases. Additionally, interpreting results requires highly skilled professionals (35).

The limitation of this study was the non-cooperation of patients in the preparation of cytology impression samples.

## Conclusion

Our present study examines the diagnostic accuracy of impression cytology in eye conjunctival lesions compared to pathology, which has a positive and significant correlation, so IC can be a helpful technique in evaluating suspected ocular surface tumors. In the case of eye conjunctival lesions, impression cytology is a relatively accurate method with appropriate sensitivity and specificity. However, it is less effective in rating the severity of dysplasia. Cytological sampling is a convenient and less invasive alternative to surgery for evaluating lesions. The method has several advantages, such as being easy, fast, cost-effective, and can be performed with local anesthesia. It can serve as a quick diagnostic technique and complementary method to histological diagnosis. Furthermore, obtaining a cytology diagnosis before surgery can aid in planning the procedure to ensure complete removal of the lesion with a free margin. This model can be further improved with the prospective use of many patients.
